# The Application of Tsallis Entropy Based Self-Adaptive Algorithm for Multi-Threshold Image Segmentation

**DOI:** 10.3390/e26090777

**Published:** 2024-09-10

**Authors:** Kailong Zhang, Mingyue He, Lijie Dong, Congjie Ou

**Affiliations:** College of Information Science and Engineering, Huaqiao University, Xiamen 361021, China; 22014082032@stu.hqu.edu.cn (K.Z.); hemingyue.xcj@foxmail.com (M.H.); dlj615846302@163.com (L.D.)

**Keywords:** tsallis entropy, long-range correlations, self-adaptive algorithm, multi-level thresholding, robustness

## Abstract

Tsallis entropy has been widely used in image thresholding because of its non-extensive properties. The non-extensive parameter *q* contained in this entropy plays an important role in various adaptive algorithms and has been successfully applied in bi-level image thresholding. In this paper, the relationships between parameter *q* and pixels’ long-range correlations have been further studied within multi-threshold image segmentation. It is found that the pixels’ correlations are remarkable and stable for images generated by a known physical principle, such as infrared images, medical CT images, and color satellite remote sensing images. The corresponding non-extensive parameter *q* can be evaluated by using the self-adaptive Tsallis entropy algorithm. The results of this algorithm are compared with those of the Shannon entropy algorithm and the original Tsallis entropy algorithm in terms of quantitative image quality evaluation metrics PSNR (Peak Signal-to-Noise Ratio) and SSIM (Structural Similarity). Furthermore, we observed that for image series with the same background, the *q* values determined by the adaptive algorithm are consistently kept in a narrow range. Therefore, similar or identical scenes during imaging would produce similar strength of long-range correlations, which provides potential applications for unsupervised image processing.

## 1. Introduction

In recent years, with the increase in digital imaging devices, image data have grown rapidly. Therefore, image processing has become more and more crucial in machine vision. During image processing, image segmentation is a fundamental step that divides an image into different regions by means of intensity, color, contour and so on. It has been successfully used in various fields [1,2,3,4] and the achievements are still growing. Technically, image segmentation mainly contains threshold-based segmentation, edge-based segmentation [5], clustering-based segmentation [6], and region-growing segmentation [7]. The threshold-based image segmentation has become the most frequently used method due to its simplicity, efficiency, and stability.

In 1980, Pun [8] first applied information entropy to image thresholding and it was improved by Kapur [9], who proposed the Maximum Shannon Entropy Thresholding algorithm. Kapur’s main idea is to treat the digital gray-level image as a matrix that contains pixels with different gray-level values. The gray-level histogram of the pixels can be considered as a kind of probability distribution. The entropy of the gray-level distribution can be maximized by a proper threshold, which is similar to maximizing the contrast between the object and the background of the image. If two or more objects exist in the same background, resulting in a multimodal gray-level distribution, the above-mentioned algorithm can be naturally extended to multi-threshold segmentation. It is worth noting that with the increasing number of thresholds, the computational complexity grows exponentially. In order to overcome this complexity and yield the optimal multi-threshold solution, swarm intelligence optimization algorithms are frequently used to solve such problems [10,11,12,13,14,15].

The concept of entropy was first proposed in thermo-statistical physics to deal with extensive systems [16]. Shannon entropy [17] inherits the extensivity and has been widely used in information theory. However, there are a lot of complex systems that present long-range interactions and the extensivities of them are broken. Extensive entropies are unsuitable to describe such systems anymore. Tsallis indicates a generalized entropic form [18] for those systems and their abnormal behaviors are well-fitted by the non-extensive parameter *q*. Over the years, Tsallis entropy has been applied not only in physics [19], but also in financial markets [20], seismology [21], bioinformatics [22], fractal networks [23], and so on. Regarding image segmentation, Tsallis entropy shows high adaptability for different types of target recognition [11,24,25] since the non-extensive parameter *q* is related to the strength of long-range correlations among image pixels. However, determining an appropriate value of *q* for a given image is still an open question in practice, since different types of images may present different patterns of correlations among pixels. Generally, the estimation of *q* values is performed empirically [26,27] in Tsallis entropy-based image segmentation, and the relationships between *q* values and the pixels’ correlations need to be further discussed.

In 2009, Rodrigues et al. [28] proposed a method to yield the optimal *q* values of images by maximizing the difference between *q*-dependent entropy and the upper limit of the histogram entropy, which sheds light on the patterns recognition of the pixels’ long-range correlations. In 2016, Ramírez-Reyes et al. [29] provided another method to obtain the non-extensive *q* values. It is based on the concept of redundancy in information theory and the entropy maximization principle, and has been successfully applied to the bi-level image thresholding [30]. While extending the bi-level segmentation to multi-level cases, with the increasing number of objects at different gray levels, the patterns of the pixels’ long-range correlation may also increase. In order to avoid the perturbations from uncertain interactions among pixels, the images generated by the unified imaging process should be adopted to illustrate the relationships between the *q* value and the long-range correlations of pixels. It is worth mentioning that in 2022, Mousavirad et al. [31] proposed a novel self-adaptive method to yield the optimal parameter *r* of Masi entropy, without relying on prior knowledge of histogram distribution or image type. This method demonstrated excellent performance in the multi-level segmentation of images with randomly natural scenes. Nevertheless, the histogram distribution may represent the correlations among pixels of an image. Therefore, it is of interest to study the relationships between the optimal entropic parameter *q* and the image histogram, which is different from the work of Mousavirad et al. [31] and can be applied to images generated by some known physical principles.

The rest of this paper is organized as follows. Section 2 briefly reviews the *q*-redundancy maximization method and introduces its application in multi-level image segmentation. In Section 3, according to the known physical principles, six image datasets are adopted for testing, and the quantitative image quality evaluation metrics such as PSNR and SSIM are introduced within multi-level image segmentation. In Section 4, the statistical results of different image datasets are illustrated so that the relationships between *q*-values and pixels’ long-range correlations are further discussed. In Section 5, the conclusions are presented.

## 2. Methods for Calculating Tsallis Entropy Index *q* and Image Segmentation

Assuming a given image size is M×N, representing the total number of pixels in it. The range of gray-level of the image is defined as i=0,1,2,…,L−1, where *L* represents the maximum gray-level of the image, such as 256. Thus, the gray-level probability distribution of the image is defined as:

(1) $$p_{i}=\frac{h_{i}}{M\times N},$$

where $h_{i}$ is the number of pixels that the gray-level value is equal to *i*, and $p_{i}\geq 0,\sum_{i=0}^{L-1}p_{i}=1$ hold. Obviously, $\left\{p_{i}\right\}$ represents the gray-level histogram distribution of the image. And Tsallis entropy is written as [18,19,24,32]:

(2) $$S_{T}=\frac{1-\sum_{i}p_{i}^{q}}{1-q},$$

where *q* is the non-extensive index.

### 2.1. Entropy Index q and the Long-Range Correlation

Ramírez-Reyes et al. suggest that each complex system has its own entropy index, and it should not be determined arbitrarily. In practice, an image can be considered a non-extensive pixel system so that the long-range correlations among them can be quantified by *q*. Therefore, two fundamental concepts, redundancy and the maximum entropy principle, play important roles in evaluating the non-extensive parameter *q*. According to the non-extensive properties of Tsallis entropy, the *q*-redundancy of an image’s histogram can be written as [29]:

(3) $$R_{T}=1-\frac{S_{T}}{S_{T\max}},$$

where $S_{T\max}=\frac{1-L^{1-q}}{q-1}$. It means that the entropy reaches its maximal at the equiprobability case, i.e., $p_{i}=p_{j}=1/L\;\left(\forall \;i,j\right)$. For a given image with a known gray-level histogram, the corresponding *q*-redundancy can be adjusted by parameter *q*. On the other hand, the histogram may exhibit long-range correlations among the pixels of the image. Therefore, maximizing the *q*-redundancy is a hopeful way to recognize the pattern of long-range correlations and yields a suitable non-extensive parameter *q*, i.e.,

(4) $$q^{*}=\arg\;\max\left(R_{T}\right).$$

With the help of $q^{*}$, the gray-level histogram of the image is re-normalized to deviate from equal probabilities as much as possible. This can result in a clearer representation of different clusters within the image, aiding in improving the quality of the image segmentation.

### 2.2. Multi-Level Thresholding Using Tsallis Entropy

Assuming that the gray-level histogram of an image is divided into m+1 parts by a set of thresholds $\vec{t}=\left(t_{1},t_{2},\ldots ,t_{m}\right)$, denoted as $\vec{C}=\left(C_{0},C_{1},\ldots ,C_{m}\right)$, after normalization, the probability distribution of each class is defined as:

(5) $$\begin{array}{c} C_{0}:\frac{p_{0}}{P_{0}},\frac{p_{1}}{P_{0}},\ldots ,\frac{p_{t_{1}}}{P_{0}}\\ \vdots \\ C_{j}:\frac{p_{t_{j}+1}}{P_{j}},\frac{p_{t_{j}+2}}{P_{j}},\ldots ,\frac{p_{t_{j}}}{P_{j}},\\ \vdots \\ C_{m}:\frac{p_{t_{m}+1}}{P_{m}},\frac{p_{t_{m}+2}}{P_{m}},\ldots ,\frac{p_{L-1}}{P_{m}} \end{array}$$

where the cumulative probabilities of m+1 categories are defined as:

(6) $$\begin{array}{c} P_{0}=\sum_{i=0}^{t_{1}}p_{i}\\ \vdots \\ P_{j}=\sum_{i=t_{j}+1}^{t_{j+1}}p_{i},\\ \vdots \\ P_{m}=\sum_{i=t_{m}+1}^{L-1}p_{i} \end{array}$$

Tsallis entropy of each region of $\vec{C}=\left(C_{0},C_{1},\ldots ,C_{m}\right)$ is obtained by the following definition:

(7) Sq0=1−∑i=0t1piP0q1−∑i=0t1piP0q(q−1)(q−1)⋮Sqj=1−∑i=tj+1tj+1piPjq1−∑i=tj+1tj+1piPjq(q−1)(q−1).⋮Sqm=1−∑i=tm+1L−1piPmq1−∑i=tm+1L−1piPmq(q−1)(q−1)

According to the pseudo-additivity property of Tsallis entropy, its multi-threshold objective function is defined as follows:

(8) $$S_{q}\left(t_{1},t_{2},\ldots ,t_{m}\right)=\sum_{i}S_{q}^{i}+\left(1-q\right)\sum_{j\neq k}S_{q}^{j}S_{q}^{k}+{\left(1-q\right)}^{2}\sum_{u\neq v\neq w}S_{q}^{u}S_{q}^{v}S_{q}^{w}+\ldots +{\left(1-q\right)}^{m}\prod_{r=0}^{m}S_{q}^{r}.$$

Maximizing the objective function $S_{q}\left(t_{1},t_{2},\ldots ,t_{m}\right)$ yields an optimal set of thresholds as follows:

(9) $${\left(\vec{t}\right)}^{*}=\arg\max\left\{S_{q}\left(t_{1},t_{2},\ldots ,t_{m}\right)\right\},$$

this algorithm is highly favored for its simplicity, intuitiveness, versatility, and excellent performance in image segmentation [33,34,35].

## 3. Image Test Sets and Quality Evaluation Parameters

There is a lot of evidence showing that parameter *q* has deep relevance with the long-range interaction in bi-level image segmentation [24,29,30,36]. However, extending bi-level thresholding to multi-level thresholding and drawing the conclusions seems not so straight. In fact, it is found that if the backgrounds of the images are of random natural scenes, the above algorithm does not exhibit significant advantages in comparison with the traditional Shannon algorithm and the original Tsallis algorithm [12]. In order to further show the relevance between pixels’ long-range correlations and nonextensivity during the imaging process, several different types of images are employed for comparison.

**Figure 1:**
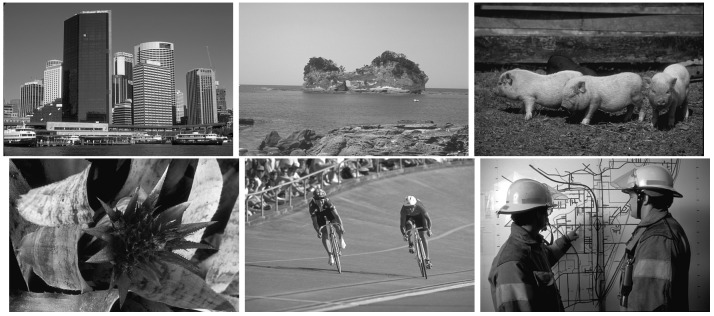
Example images from the BSDS0500 image dataset.

BSDS0500 is an image dataset consisting of randomly natural scenes. This dataset contains 500 images taken from real-world natural scenes, covering a variety of views and objects, including but not limited to modern urban landscapes, natural landscapes, animals and plants, human activities, and so on. These images provide diverse scenes and various visual information. Here, are a few examples from this dataset (Figure 1).

INFRAIMGS1, INFRAIMGS2, INFRAIMGS3, and INFRAIMGS4 are series of image datasets containing lots of infrared images captured by fixed infrared cameras at different moments. These datasets record specific activities and movements of objects in different scenes.

- INFRAIMGS1: These images capture the activities of pedestrians and vehicles on two fixed outdoor road scenes. The dataset consists of 464 images extracted from frames, with a resolution of 550×365.
- INFRAIMGS2: This dataset depicts student activities at a fixed intersection near a teaching building. It comprises 264 images extracted from frames, with a resolution of 320×240.
- INFRAIMGS3: Presenting scenes fixed inside a cabin, focusing on the movement of individuals in the area. This dataset contains 253 images extracted from frames, capturing scenes of interaction and movement between individuals, with a resolution of 320×240.
- INFRAIMGS4: Showcasing scenes fixed in squares or similar open spaces, capturing pedestrians engaged in activities such as running, walking, or other leisure activities. The dataset comprises 118 images extracted from frames, with a resolution of 360×240.

CTIMGS is a collection of medical chest CT images covering scans of chests from different patients. These images have a fixed black background, and the dataset comprises a total of 600 images, with a resolution of 224×224. Below are examples of images from these datasets.

**Figure 2:**
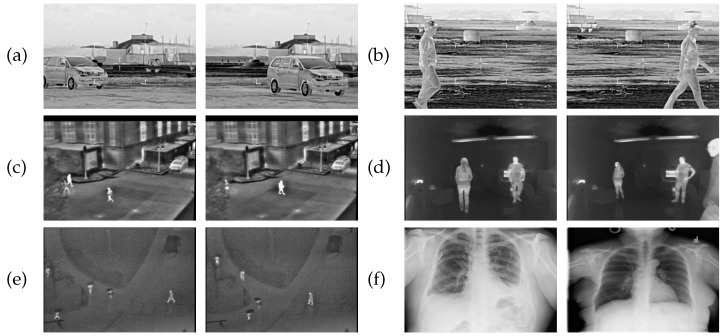
(**a**,**b**) are images in the image set INFRAIMGS1. (**c**) are images in the image set INFRAIMGS2. (**d**) are images in the image set INFRAIMGS3. (**e**) are images in the image set INFRAIMGS4. (**f**) are images in the image set CTIMGS.

In the same dataset of Figure 2, those images are taken from the same background and generated by the same imaging principle, i.e., infrared imaging for INFRAIMGS1-4 and X-ray imaging for CTIMGS. These specified types of images can help us to further understand the pixels’ long-range correlations in the imaging stage.

**Figure 3:**
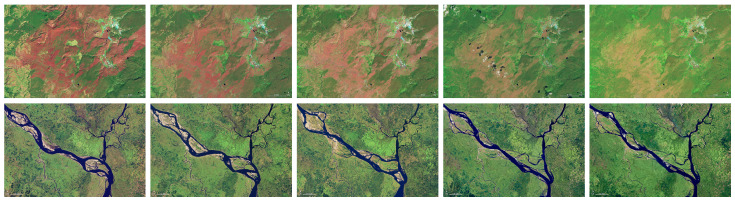
Example satellite images of Yellowstone and Padma. In the first row, from **left** to **right**, they are Yellowstone 1993, 1997, 2002, 2009, and 2017. In the second row, from **left** to **right**, they are Padma 1992, 1996, 2004, 2014, and 2016.

The images shown in Figure 3 are generated by satellite remote sensing that captures changes in the Yellowstone and Padma regions over many years, with a resolution of 720×480.

In order to evaluate the effectiveness of this self-adaptive multi-level segmentation algorithm, PSNR (Peak Signal-to-Noise Ratio) and SSIM (Structural Similarity Index) are adopted as quality indices. PSNR [37] represents the ratio of the peak signal to the noise. In image multiple-thresholding due to the gray-level compression, the output image is generally different from the original one. PSNR can precisely measure this difference and it is defined as:

(10) $$PSNR=10\log_{10}\left(\frac{255^{2}}{MSE}\right),$$

MSE in Equation (10) is the mean squared error between the output image and the input image, and 255 is the maximum gray-level value in the image in general. MSE can be written as:

(11) $$MSE=\frac{1}{M\times N}\sum_{i=1}^{M}\sum_{j=1}^{N}{\left[I\left(i,j\right)-K\left(i,j\right)\right]}^{2},$$

where $I\left(i,j\right)$ and $K\left(i,j\right)$ represent the original image and the image after segmentation, respectively.

The typical PSNR values in image segmentation range from 10 dB to 50 dB [38]. A higher PSNR value indicates a smaller distortion in the output image and a higher quality of segmentation. PSNR closing to 50 dB indicates that the segmented image has very minor errors. If PSNR is greater than 30 dB, it is difficult for the human eyes to perceive differences between the segmented image and the original one. For PSNR ranging from 20 dB to 30 dB, the differences become noticeable to the human eyes. In the range of 10 dB to 20 dB, the differences become larger. Nevertheless, the human eyes can still recognize the main structures in the output image. If PSNR is below 10 dB, it becomes challenging for humans to determine if there are any correlations between the input and output images. PSNR is currently the most frequently used objective measure for evaluating image quality. However, many experimental results have shown that PSNR scores may not fully coincide with the visual quality perceived by the human eyes. It is possible for images with higher PSNR to appear worse in visual quality than those with lower PSNR scores since the human visual system’s sensitivity to errors is affected by a lot of factors that are more complicated than Equation (10).

SSIM [39] is another quality metric that measures the similarity between two digital images. The recognition criteria of the human visual system, such as luminance, contrast, and structural information, are taken into account [40,41] to yield the expression of SSIM as:

(12) $$SSIM\left(x,y\right)=\frac{\left(2\mu_{x}\mu_{y}+C_{1}\right)\left(2\sigma_{xy}+C_{2}\right)}{\left(\mu_{x}^{2}+\mu_{y}^{2}+C_{1}\right)\left(\sigma_{x}^{2}+\sigma_{y}^{2}+C_{2}\right)},$$

where *x* and *y* represent the images before and after segmentation, $\mu_{x}$ and $\mu_{y}$ denote the mean intensity of the corresponding images, $\sigma_{x}^{2}$ and $\sigma_{y}^{2}$ represent the standard deviations respectively, $\sigma_{xy}$ denotes the covariance of the images before and after segmentation, $C_{1}$ and $C_{2}$ are two constants to avoid zeros appearing in the denominator. Equation (12) shows that SSIM is a dimensionless value between 0 and 1, where smaller differences between the original and segmented images yield closer value to 1. Due to its simplicity and effectiveness, SSIM has been widely used in various applications related to image and video processing in recent years, such as image compression [42], image watermarking [43], wireless video streaming [44], and magnetic resonance imaging [45].

In practice, PSNR is more sensitive to additive Gaussian noise, while it exhibits lower sensitivity to JPEG compression. Conversely, SSIM is more sensitive to JPEG compression but relatively less responsive to additive Gaussian noise [37]. Therefore, we employ both PSNR and SSIM to assess the quality of the self-adaptive multi-level segmentation.

## 4. Experimental Results and Discussion

In order to show the detailed relevance between pixels’ long-range correlations and non-extensive entropy index *q* within a multi-level segmentation case, Shannon entropy and traditional Tsallis entropy are adopted as benchmarks to show the performance of the proposed self-adaptive algorithm. As mentioned above, Shannon entropy neglects the long-range correlations among image pixels and shows the extensive property. Tsallis entropy generalized the application scope of Shannon entropy by linking the strength of long-range correlations to non-extensive index *q*.

Images from the six datasets mentioned in Section 3 are processed by using three different multi-level segmentation algorithms, i.e., Shannon, and Tsallis (*q* = 0.8), and proposed, to yield the optimal results, respectively.

**Figure 4:**
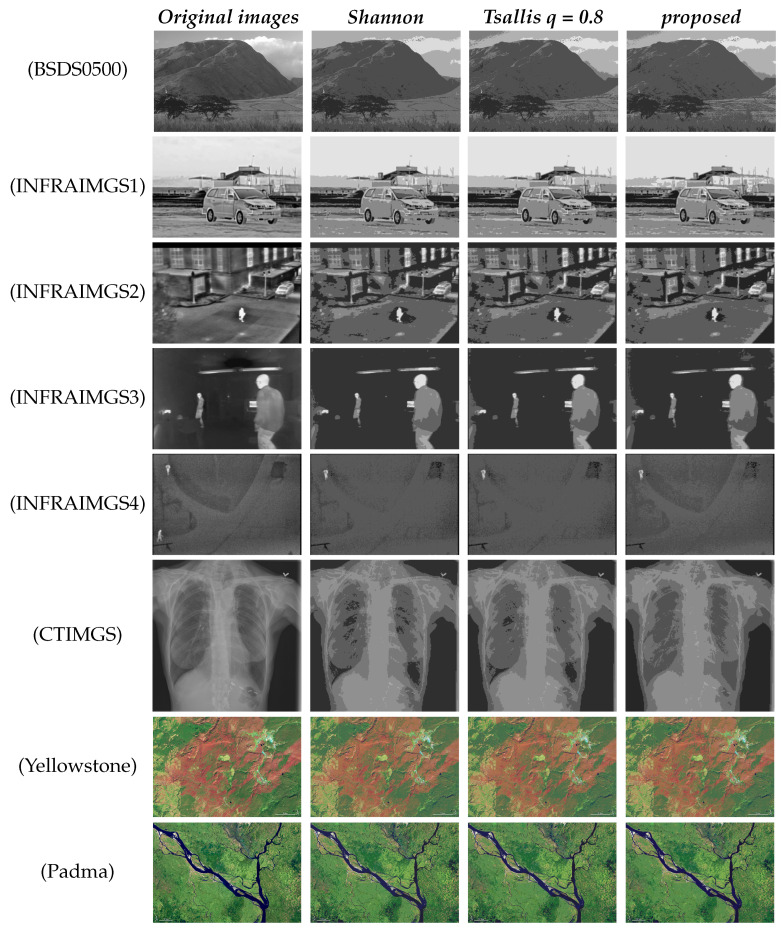
The four-level segmentation results of the typical images from eight datasets by using three different algorithms.

Figure 4 shows the four-level segmentation results of sample images from eight datasets. For the image from BSDS0500, the result of the Shannon algorithm looks closest to the original image, which indicates that the long-range correlations among image pixels can be neglected. Since the images in BSDS0500 are generated from random scenes, it is inadequate to say that the pixels of different images should always exhibit a long-range correlation. However, for images from the other seven datasets, the segmentation results of the proposed algorithm consistently show the superiority to the other two algorithms. Images in INFRAIMGS1-4 are generated by infrared cameras so infrared radiation plays an important role during the imaging process. It is well known that infrared radiation depends closely on the temperature of the objects. Therefore, the patterns of pixels’ long-range correlations actually reflect the temperature distributions of different objects in infrared images. This kind of long-range correlation can be successfully captured by the self-adaptive multi-level segmentation algorithm, which is more flexible than the traditional Tsallis entropy with a fixed *q* index. The evidence can also be found in the dataset of medical CT images, in which the pixels’ gray-level directly depend on the absorption of X-rays by different organs inside human body. Therefore, the gray-level values of pixels belonging to the same organ should have strong correlations and it is suitable to describe this kind of correlation by adaptive *q* index rather than fixed *q*. Moreover, the proposed algorithm is also effective to color images since color image can be considered as a combination of three colors (red, green, and blue), and the strength distribution of each color is similar to that of gray-level image. In fact, the satellite remote sensing images record the geography information of the earth’s surface so that the pixels belong to the same landform are correlated.

In order to show the segmentation results quantitatively, the output images of the three algorithms are compared with the corresponding original images in terms of PSNR and SSIM. Table 1 shows part of the PSNR results of BSDS0500 images by using Shannon, Tsallis (*q* = 0.8), and the proposed muti-level thresholding algorithms, in which the number of thresholds is 4. In each line of Table 1, the maximum PSNR value (with bold font) indicates that the corresponding algorithm is the most suitable one for the image named at the beginning of the line.

Therefore, for all 500 images of BSDS0500, one can statistically obtain the most suitable rates of three algorithms. They are 32% for the Shannon algorithm, 24.6% for the Tsallis algorithm with *q* = 0.8, and 55% for the proposed self-adaptive algorithm. It is worth mentioning that the sum of the above three most suitable rates slightly exceeds 100% because there are a few images, such as BSDS00109 in Table 1, that happen to obtain the same best result by using different algorithms. Nevertheless, the probability of such a case is small so that the images in the other five datasets can be processed in the same way. The statistical results are shown in Table 2.

Interestingly, unlike the distribution of the most suitable rates in BSDS0500, all the other five datasets show a notable tendency (the corresponding rates are far larger than 65%) to the proposed algorithm. Especially for INFRAIMGS2, all of the 264 images in it recognize the self-adaptive *q* as the most suitable values to present the pixels’ long-range correlations. The experimental results also show that the distribution of 264 *q* values ranges from 0.490 to 0.513, a very small interval. In fact, all images in INFRAIMGS2 have the same background and the ratios of the foreground (moving objects) to the full image size are small. It is reasonable to say that the strength of long-range correlations in each image of this dataset should be quite similar, but cannot be empirically determined by a fixed value. Other datasets that have the same characteristics with INFRAIMGS2, all exhibit the consistency in the range of *q*, such as 0.512≤q≤0.602 for INFRAIMGS1, 0.509≤q≤0.580 for INFRAIMGS3, 0.381≤q≤0.512 for INFRAIMGS4. These behaviors coincide with the imaging principles mentioned above.

The validity of self-adaptive *q* can be further confirmed by SSIM. Table 3 shows part of the SSIM results for the same images adopted in Table 1 by using three different multi-level thresholding algorithms, in which the number of thresholds is still 4. Since the definition of SSIM is totally different from that of PSNR, their responses to the same output image may not always be consistent with each other. Such as BSDS00116, according to the results of PSNR, Tsallis algorithm with *q* = 0.8 is suggested as the most suitable one, while Shannon algorithm yields the highest SSIM score. Nevertheless, the statistical results of the most suitable rates suggested by SSIM can also be obtained in the same way as Table 2, and they are shown in Table 4.

The sum of the most suitable rate for each dataset also slightly exceeds 100%, and the reason is similar to that in Table 2. It is found that all of the infra-image datasets show their preferences for the adaptive *q* as a measure of the strength of pixels’ long-range correlations under the criterion of SSIM. The statistical result of INFRAIMGS2 is the most notable one. All of the images in it adopt the proposed algorithm to achieve the highest scores defined by not only PSNR but also SSIM. Besides Table 2 and Table 4, the results of the most suitable rates over six datasets can be extended to the cases of a larger number of thresholds, as shown in Table 5, Table 6, Table 7 and Table 8.

Table 5 and Table 6 list the most suitable rates for the five-level segmentation of different datasets, where PSNR and SSIM are adopted as the criteria, respectively. And increasing the number of thresholds from 5 to 6, the results are listed in Table 7 and Table 8. Impressively, images of INFRAIMGS2 show their robust preferences for the proposed algorithm in spite of the increasing number of thresholds. This kind of robustness can also be found in other datasets, such as INFRAIMGS1, INFRAIMGS4, CTIMGS. Therefore, it is suitable to adopt the self-adaptive *q* value to measure the strength of long-range correlations within images generated by known physical principles. In other words, the physical properties of objects shown in the images can be connected to the non-extensive parameter *q* by maximizing the redundancy of the histogram distribution. It is worth mentioning that for INFRAIMGS3, the most suitable rate of proposed algorithm yields by PSNR keep decreasing when the number of thresholds grows. Since the gray-level gradations of images in INFRAIMGS3 are not plentiful, the increasing number of thresholds may lead to over-segmentation and the results evaluated by PSNR and SSIM become unstable. Nevertheless, in most cases, the proposed algorithm shows effectiveness (with the most suitable rate higher than 65%) and robustness (keeps fixed when the number of thresholds increases) in automatically detecting the long-range correlations among pixels of infrared images and medical images.

In Table 9, we compare the PSNR and SSIM results of multi-level segmentation for images of Figure 3 by using the same algorithms of Table 1, Table 2, Table 3, Table 4, Table 5, Table 6, Table 7 and Table 8. The results clearly show that the proposed self-adaptive algorithm consistently performs the best in most cases. Therefore, using the self-adaptive algorithm can more accurately capture the long-range correlation strength of surface features in satellite remote sensing images. This indicates that the proposed self-adaptive algorithm is suitable not only for grayscale images but also for color images that are generated by known physical principles.

**Figure 5:**
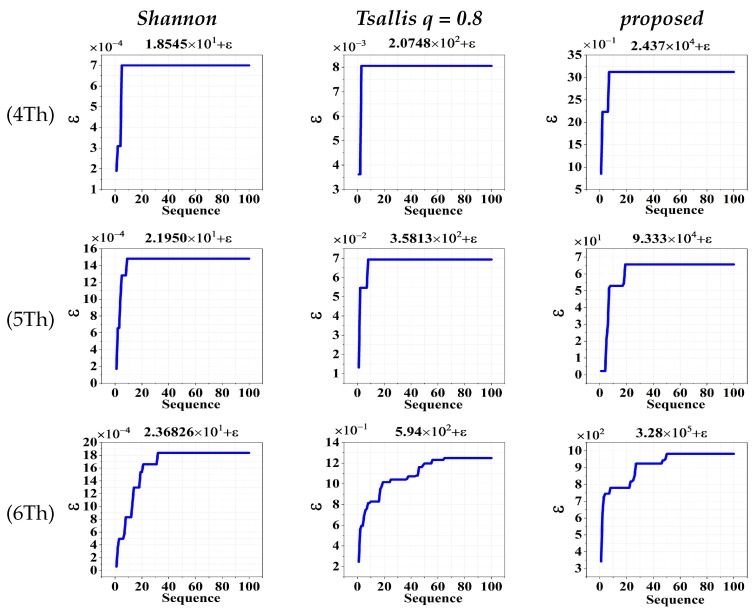
Sorted fitness values for INFRAIMGS2 from Figure 4, based on 100 independent runs by different algorithms with different number of thresholds.

In order to evaluate the robustness of three algorithms in different threshold levels, we randomly adopted an image to yield the optimal fitness values for comparison. For a given number of thresholds, each algorithm is independently applied to the image for 100 runs. Since the idea of swarm intelligence is included in the algorithm, the corresponding 100 results are stochastic to some extent. Nevertheless, the results can be sorted in ascending order and plotted with the rearranged sequence, as they are shown in Figure 5. Clearly, for different algorithms with different numbers of thresholds, all of the plotted results have a flat and long tail. It means that the highest fitness value can be reproduced a lot of times over 100 stochastic runs. Therefore, the above-mentioned algorithms are robust enough to yield reliable results.

## 5. Conclusions

In image segmentation, determining the non-extensive parameter *q* of Tsallis entropy is an intriguing task. Since the value of *q* represents the strength of long-range interactions among pixels of the images that are generated by some known physical principles, it cannot be determined empirically. In the present paper, with the help of maximizing *q*-redundancy, we further study the connections between the physical properties of objects shown in the images and the self-adaptive value of *q* in multi-threshold image segmentation. In comparison with the Shannon entropy algorithm and the traditional Tsallis entropy algorithm with *q* = 0.8, it is found that the self-adaptive algorithm shows high effectiveness and robustness to infrared images, medical CT images, and color satellite remote sensing images. The superiority and consistency of the present algorithm are qualitatively illustrated by means of PSNR and SSIM when the number of thresholds is set as 4, 5, and 6, respectively. In addition, for a series of images generated by the same process and sharing the same background, the long-range correlation pattern among pixels should be quite similar. The self-adaptive *q* values of those images are also quite similar, as expected. All of these advantages will be helpful for the further applications of Tsallis entropy in multi-level image segmentation.

## Figures and Tables

**Table 1:** Part of PSNR results for images in BSDS0500 with different four-level thresholding algorithms.

	*Shannon*	*Tsallis q = 0.8*	*Proposed*
BSDS00065	27.7979	27.8888	**28.0085**
BSDS00109	**28.0865**	**28.0865**	27.4921
BSDS00116	26.9156	**26.9787**	26.9286
BSDS00203	**29.1844**	29.0696	29.1492
BSDS00474	**29.5335**	29.3923	23.0752

Bold font refer to the best results.

**Table 2:** The most suitable rates of three algorithms suggested by PSNR for six datasets when the number of thresholds is 4.

	*Shannon*	*Tsallis q = 0.8*	*Proposed*
BSDS0500	32%	24.6%	55%
INFRAIMGS1	7.9%	13.1%	**84.3%**
INFRAIMGS2	0%	0%	**100%**
INFRAIMGS3	7.9%	13.8%	**87.3%**
INFRAIMGS4	14.1%	16.3%	**73.7%**
CTIMGS	14.1%	15.6%	**75.8%**

Bold font refer to the rates larger than 65%.

**Table 3:** Part of SSIM results for images in BSDS0500 with different four-level thresholding algorithms.

	*Shannon*	*Tsallis q = 0.8*	*Proposed*
BSDS00065	**0.7484**	0.7466	0.7374
BSDS00109	**0.6423**	**0.6423**	0.6383
BSDS00116	**0.7257**	0.7237	0.7181
BSDS00203	0.7199	0.7185	**0.7205**
BSDS00474	**0.8066**	0.8063	0.7166

Bold font refer to the best results.

**Table 4:** The most suitable rates of three algorithms suggested by SSIM for six datasets when the number of thresholds is 4.

	*Shannon*	*Tsallis q = 0.8*	*Proposed*
BSDS0500	36.8%	23.2%	52.6%
INFRAIMGS1	12.7%	19.8%	**77.6%**
INFRAIMGS2	0%	0%	**100%**
INFRAIMGS3	10.6%	19.3%	**78.6%**
INFRAIMGS4	6.7%	16.9%	**89.8%**
CTIMGS	29.5%	25.8%	53.5%

Bold font refer to the rates larger than 65%.

**Table 5:** The most suitable rates of three algorithms suggested by PSNR for six datasets when the number of thresholds is 5.

	*Shannon*	*Tsallis q = 0.8*	*Proposed*
BSDS0500	32.6%	28.2%	39.2%
INFRAIMGS1	21.3%	38.8%	**66.2%**
INFRAIMGS2	0%	0%	**100%**
INFRAIMGS3	19.3%	32.8%	53.4%
INFRAIMGS4	6.7%	16.9%	**89.8%**
CTIMGS	14.7%	15.7%	**75%**

Bold font refer to the rates larger than 65%.

**Table 6:** The most suitable rates of three algorithms suggested by SSIM for six datasets when the number of thresholds is 5.

	*Shannon*	*Tsallis q = 0.8*	*Proposed*
BSDS0500	34.6%	27.6%	50%
INFRAIMGS1	11.9%	22.2%	**87.1%**
INFRAIMGS2	0%	0%	**100%**
INFRAIMGS3	2.3%	8.6%	**93.3%**
INFRAIMGS4	2.5%	8.4%	**94.9%**
CTIMGS	30%	28.2%	50.3%

Bold font refer to the rates larger than 65%.

**Table 7:** The most suitable rates of three algorithms suggested by PSNR for six datasets when the number of thresholds is 6.

	*Shannon*	*Tsallis q = 0.8*	*Proposed*
BSDS500	32%	30.2%	52.2%
INFRAIMGS1	20.9%	37.7%	**66.8%**
INFRAIMGS2	0%	0%	**100%**
INFRAIMGS3	40.7%	25.2%	40%
INFRAIMGS4	5.9%	11%	**89.8%**
CTIMGS	13.3%	18.6%	**74.5%**

Bold font refer to the rates larger than 65%.

**Table 8:** The most suitable rates of three algorithms suggested by SSIM for six datasets when the number of thresholds is 6.

	*Shannon*	*Tsallis q = 0.8*	*Proposed*
BSDS0500	38.4%	27.6%	48.8%
INFRAIMGS1	11.8%	21.7%	**87.1%**
INFRAIMGS2	0%	1.9%	**98.1%**
INFRAIMGS3	18.9%	32.4%	56.6%
INFRAIMGS4	5%	10.1%	**91.5%**
CTIMGS	27.5%	24%	56.2%

Bold font refer to the rates larger than 65%.

**Table 9:** Comparisons of PSNR and SSIM values for multi-level segmentation of color images by using different thresholding algorithms.

Image	Thresholds	*Shannon*			*Tsallis q = 0.8*			*Proposed*	
		PSNR	SSIM		PSNR	SSIM		PSNR	SSIM
	4	26.0698	0.6961		26.0703	0.6942		**26.2653**	**0.7047**
Yellowstone1993	5	26.6900	0.7251		26.8004	0.7330		**27.0259**	**0.7458**
	6	27.8027	0.7807		27.8086	0.7809		**27.9138**	**0.7837**
	4	26.4339	0.6099		26.4485	0.6115		**26.5099**	**0.6188**
Yellowstone1997	5	27.1560	0.6495		27.1599	0.6494		**27.2653**	**0.6663**
	6	28.5670	0.7471		28.5180	0.7429		**29.1877**	**0.7690**
	4	25.9700	0.6461		26.0475	0.6488		**26.2045**	**0.6615**
Yellowstone2002	5	27.7278	0.7475		27.9106	**0.7556**		**27.9259**	0.7553
	6	28.0986	0.7670		28.0818	0.7648		**28.8121**	**0.7892**
	4	26.1092	0.5872		26.1376	**0.5884**		**26.2793**	0.5857
Yellowstone2009	5	26.3475	0.6165		26.4753	0.6186		**26.5855**	**0.6251**
	6	28.6586	0.7335		28.7034	0.7324		**28.7736**	**0.7370**
	4	28.1074	0.6774		28.0817	0.6772		**28.2040**	**0.6882**
Yellowstone2017	5	28.8943	0.7158		28.9058	0.7164		**29.0543**	**0.7274**
	6	30.5397	0.7858		30.5387	0.7858		**30.5521**	**0.7876**
	4	25.6878	0.7624		25.7831	0.7648		**25.8479**	**0.7659**
Padma1992	5	27.1510	0.8105		27.3246	0.8185		**27.3953**	**0.8205**
	6	28.2147	0.8488		28.3376	0.8550		**28.5972**	**0.8680**
	4	26.1716	0.7855		26.2547	0.7904		**26.2882**	**0.7920**
Padma1996	5	27.0581	0.8200		27.4121	**0.8321**		**27.4812**	0.8316
	6	28.3429	0.8548		28.4055	0.8583		**28.5049**	**0.8631**
	4	26.0027	0.7803		26.0819	0.7841		**26.1430**	**0.7880**
Padma2004	5	27.5688	0.8339		27.6402	0.8351		**27.7453**	**0.8407**
	6	28.0786	0.8497		28.1412	0.8532		**28.1771**	**0.8538**
	4	25.2863	0.8131		25.6776	**0.8206**		**25.8834**	0.8158
Padma2014	5	26.5513	0.8528		26.9556	**0.8539**		**27.0405**	0.8399
	6	27.9148	0.8742		27.9757	0.8762		**28.5761**	**0.8884**
	4	24.9751	0.7897		25.1864	0.7925		**25.4546**	**0.7933**
Padma2016	5	25.9929	0.8145		26.1939	0.8184		**26.3677**	**0.8220**
	6	27.6637	0.8668		27.8477	0.8705		**28.1865**	**0.8790**

Bold font refer to the best results.

## Data Availability

The original contributions presented in the study are included in the article, further inquiries can be directed to the corresponding author.
